# Commentary: How do we get platforms to share data with independent researchers? Regulation alone will not cut it: a commentary on Livingston et al. (2023), Bourgaize et al. (2025)

**DOI:** 10.1111/camh.70020

**Published:** 2025-07-20

**Authors:** Andreu Casas, Georgia Dagher, Ben O'Loughlin

**Affiliations:** ^1^ Department of Politics, International Relations and Philosophy, New Political Communication Unit Royal Holloway University of London London UK

**Keywords:** data access, online safety, Social media

## Abstract

We respond to articles in the *Child and Adolescent Mental Health* journal about whether, and under what, conditions researchers should collaborate with digital companies. In particular, we discuss the challenges academics face to access and study platform data. Independent academic research in this area is crucial for identifying and combating any potential negative effects that platforms can have on individuals and societies. Past discussions on academic data access have focused on platform regulation and data governance. However, in this commentary, we argue that even if key stakeholders agree on a regulatory and governance model, platforms have strong incentives to not comply—or to comply only partially. We advocate for a more holistic strategy aiming at influencing regulation, public opinion, news media, diverse political groups and for building a robust oversight structure.

Policymakers and the public are increasingly concerned about the dangers of social media for individuals and society—especially as some large platforms are making political alliances in an antiregulation front, growing more powerful and less transparent. Many have discussed the perils of social media for people's mental wellbeing, particularly young adults. In a recent report (Casas, Dagher, & O'Loughlin, 2025), we emphasise platform threats to *political online safety* and democracy, including the spread of dis/misinformation, hate speech and election interference operations.

A challenge for independent academic research in this area is that the platforms own and control the data needed for conducting key analytical studies. In previous debate pieces published in the *Child and Adolescent Mental Health* journal, Livingstone, Orben, and Odgers (2023) discuss whether and under what conditions researchers should collaborate with digital companies in analysing company data, and Bourgaize et al. (2025) discuss some practical guidance for informing researchers about when it might be (not) advisable to enter into such partnerships. In the report mentioned above (Casas et al., 2025), we also call for better data access for academics: independent analysis of platform data is a must if we are to truly understand online threats and how to address them. For example, to what politically relevant content are users exposed? Is such content curated by the user or by platform algorithms? Who authored the content? Does it contain toxic speech or misinformation? Does it shape the attitudes or behaviour of the user? A clear data trail from cause to effect is blocked from researchers. In the report, we also discuss extensively several key values and principles that we argue should govern academic access to platform data.

Unfortunately, platforms have strong incentives to *not* share data with independent researchers, or to instead share curated, biased and/or incomplete data. Toxic content, such as misinformation, often generates higher engagement levels (Vosoughi, Roy, & Aral, 2018), positively contributing to the platforms' revenues. Thus, research uncovering negative societal effects can seriously damage the platforms' reputation and prospects. Additionally, independent researchers and the public at large suffer from a clear information asymmetry, where only platforms know what data they possess as well as the ways in which their recommendation and content moderation algorithms operate.

For example, some have noted that Meta tweaked its algorithms during the 2020 US election period, potentially affecting the results of studies about the impact of its algorithm on election‐related information consumption (US 2020 Election Research Project), which were being carried out by the platform itself in partnership with independent researchers (Bagchi et al., 2024). Others have flagged inaccuracies in data provided by TikTok for academic research (Pearson et al., 2024). Moreover, platforms can revoke data access to independent researchers at will at any time. X discontinued the Academic Application Programming Interface (API) in June 2023, and Meta shut down CrowdTangle (a tool widely used by independent researchers) in August 2024.

This speaks directly to some of the points raised by Bourgaize et al. (2025). Although research partnerships such as Meta's US 2020 Election Research Project can potentially yield unprecedented scientific insights, it is difficult to find convincing accounts where that has indeed been the case. Also, as discussed by Bourgaize et al. (2025), in making the decision about whether to directly partner with digital companies, researchers should definitely conduct due diligence checks and consider past platform actions in the process, including the ones described above.

The debate hence on how to govern academic access to platform data is an important one. However, even if independent researchers, policymakers and regulators (and potentially platforms, at least in principle) agree on a governing model, *how do we get platforms to actually deliver*? We argue that a holistic approach that takes legislation, oversight, politics, public opinion and new media into account is needed.

## Legislation

We need clear mandates for platforms specifying what data they must provide to independent researchers and under what conditions. In our previous report, we provided very detailed ideas regarding data needs and governance (Casas et al., 2025). Article 40 of the EU Digital Services Act (DSA) is a great starting point, yet it only applies to research focusing on the systemic risks in the European Union identified in Art. 34, such as the dissemination of illegal content, and potential negative effects on fundamental rights, civic discourse and elections, public security and the physical and mental wellbeing of citizens, particularly minors. Additionally, secondary legislation is still needed for fully rolling out and speeding up crucial processes of the DSA. Similar (and harmonised) legal prerogatives are needed worldwide to ensure that academics across the globe can access platform data and that a diversity of viewpoints contribute to this research. International professional associations (such as the Coalition for Independent Technology Research and the European Digital Media Observatory) and international organisations (such as UNESCO) can play a key role in fostering policy debates, putting forward expert recommendations and pushing for harmonised regulations. Yet, it will come down to national parliaments with actual enforcing powers to enact such legislation.

## Oversight

*Legislation alone won't cut it*. As mentioned, platforms have incentives to *not* share (accurate nor complete) data with independent researchers. Additionally, there is a clear information asymmetry—with platforms having the monopoly on knowing what data they exactly have in their possession. Past platform actions mean that trust in platforms among the research community is at a very low point. To address this, strong mechanisms must be in place to monitor platforms' compliance with academic data‐access prerogatives. Here, the literature on algorithmic accountability and algorithmic auditing (Vecchione, Levy, & Barocas, 2021) can be of particular relevance. An idea could be to provide direct onsite access to a selected number of independent researchers solely for the purpose of assessing the completeness of the data provided by platforms. For example, in a research collaboration with Meta set up by Social Science One, a Commission of respected scholars oversaw and participated in the data curation process by ‘*receiv[ing] access to the company's operations and necessary data under confidentiality agreements*’ (King & Persily, 2019, p. 3). As already identified by the promoters of that initiative, this can only work if such a commission ‘*has the trust of the broader academic community and general public*’ (p. 4). Another idea could be to create and support a specialised unit in charge of monitoring the quality of the data provided by the platforms. This could entangle a variety of techniques, such as systematically comparing the information in the provided datasets to the data one can see in the platform (Pearson et al., 2024), deploying controlled accounts in the platform for which data is known and can be easily validated, and independently track platform actions (and potential biases) on a relevant subset of accounts.

## Politics

Organisations in charge of managing and overseeing academic access to platform data should also actively work with policymakers in building a political consensus around this issue. Given the societal prevalence and relevance of platforms, we would all benefit from independent scrutiny of platform actions and consequences. Unfortunately, in recent years, there has been a rise in an antiplatform regulation discourse, which can prevent progress in the much‐needed legislation and regulation described above. The research community must devote efforts to communicate their needs to political forces across the ideological aisle and to address any concerns particular political groups might have regarding academic independent oversight of platform actions.

## Public opinion

Similarly, platform regulation is an increasingly politicised and polarised topic among the public. An increase in public support for academic access and analysis of platform data will not only put more pressure on politicians to take action, but also on platforms. We need more work investigating how citizens form opinions about tech companies, platforms, algorithms, and the need to regulate them. Additionally, we need to explore in more detail what kind of language works best at communicating, to a wider audience, the potential societal threats of social media and the need for independent scrutiny—as there is yet no academic consensus.

## News media

Mass media plays a key role in setting political and public agendas, and in framing political debates. Media attention is a scarce resource, with a limited number of topics competing for attention in the media agenda at any given point in time. Journalists follow their own principles and values when determining the newsworthiness of topics and events—as do other kinds of opinion leaders such as influencers. Independent platform researchers must develop a coordinated communication strategy to ensure a persistent and consistent media presence, in order to communicate the urgency of independent scrutiny of platforms to policymakers and the public. Building on the previous point, this requires identifying: the kinds of language that work for each target audience, as well as the person/s and channel that are best suited to deliver the message to that particular audience. The academic community should aim to find an inclusive forum where this communication strategy can be discussed—such as international conferences and gatherings of professional organisations; and then aim to promote it at each national and institutional level.

Platforms have little incentive to offer researchers access to data. To them, it is *their* data, to be used to maximise economic profit for as long as possible. We have discussed here that there are levers, public and private, that we can use to alter the equation. We are not saying that this is an easy task—on the contrary. But what matters is that these levers exist and that we can use them, but that doing so must be a coordinated effort across legislation, oversight, politics, public opinion, and news media. This takes the will and organisational skill of all of us, across sectors and countries. The failure so far of legislation and regulation alone is evident. We present this joint strategy as the basis for a coordinated push to open up data access, and encourage readers and the community to contribute to this debate.

## Conflict of interest statement

The authors have declared that they have no competing or potential conflicts of interest.

## Funding information

AC acknowledges the support of research grants from NWO‐VENI (VI.Veni.211R.052), Horizon Europe (101177574), and a Social Purpose grant from Royal Holloway University of London.

## Ethics statement

Not applicable to this Commentary article.

## Data Availability

Data sharing is not applicable to this article as no new data were created or analysed.
